# McMYB10 Modulates the Expression of a Ubiquitin Ligase, McCOP1 During Leaf Coloration in Crabapple

**DOI:** 10.3389/fpls.2018.00704

**Published:** 2018-06-04

**Authors:** Ke-Ting Li, Jie Zhang, Yan-Hui Kang, Meng-Chen Chen, Ting-Ting Song, Hui Geng, Ji Tian, Yun-Cong Yao

**Affiliations:** ^1^Department of Plant Science and Technology, Beijing University of Agriculture, Beijing, China; ^2^Key Laboratory of New Technology in Agricultural Application of Beijing, Beijing University of Agriculture, Beijing, China; ^3^Beijing Collaborative Innovation Center for Eco-Environmental Improvement with Forestry and Fruit Trees, Beijing, China

**Keywords:** *McMYB10*, *McCOP1*, self-regulation, anthocyanins, *Malus* crabapple

## Abstract

In higher plants, anthocyanins are protective secondary metabolites, which contribute to the color of leaves, stems, flowers, and fruits, and have been found to have an antioxidant role in human health. In this study, we determined the expression of *McMYB10* and its specific E3 ubiquitin ligase, McCOP1, in crabapple leaves during the course of a day and in five leaf development stages. Interestingly, the results showed that the transcription level of *McCOP1* genes was higher in daylight than at night, and the transcripts of *McMYB10* presented a positive correlation with the transcription of *McCOP1-1* and *McCOP1-2* and anthocyanin accumulation in a crabapple cultivar with red-colored leaves. Several MYB transcription factor (TF) binding sites of the MYBCORE type were found in the *McCOP1-1* and *McCOP1-2* promoters, and we deduced that there may be a relationship between *McMYB10* and *McCOP1-1* and *McCOP1-2* at the transcriptional level. Yeast one hybrid (Y1H) and electrophoretic mobility shift assays (EMSA) demonstrated that the *McMYB10* TF binds specifically to the promoter of *McCOP1-1* and *McCOP1-2*. Furthermore, increased levels of McMYB10 promoted anthocyanin biosynthesis and the expression level of *McCOP1-1* and *McCOP1-2* in crabapple leaves during continuous light treatments, and overexpression or silencing of *McMYB10* in crabapple leaves and apple fruits also result in an increase or decrease, respectively, in the expression of *McCOP1-1* and *McCOP1-2* and in anthocyanin biosynthesis. Our results reveal a new self-regulation mechanism in where *McMYB10* modulates its own expression by activating *McCOP1-1* and *McCOP1-2* expression to promote ubiquitination of the McMYB10 protein by McCOP1.

## Introduction

Anthocyanins are a class of flavonoid pigments that are responsible for the characteristic red, blue or purple colors of many organs and tissues in a wide range of plant species (Field et al., 2001; Honda and Saito, 2002). The important roles of anthocyanins include that they protect against overexposure to ultraviolet light and extreme temperatures, enhancing resistance to biotic stress and attracting pollinators and seed dispersers (Simmonds, 2003; Treutter, 2005; Page et al., 2012). Moreover, anthocyanins are believed to provide many health benefits, such as reducing the risk of a series of health problems, including diabetes, heart disease, cancer and degenerative conditions (Zhang et al., 2008; Sun et al., 2013).

Anthocyanins are biosynthesized by a common branch of the flavonoid biosynthetic pathway and have been widely studied in several plant species, including *Arabidopsis thaliana*, *Petunia hybrida*, *Zea mays*, *Vitis vinifera*, and *Malus domestic* (Kobayashi et al., 2002; Chagné et al., 2013; Litchfield, 2013; Misyura et al., 2013; Cavallini et al., 2014). The structural genes for anthocyanin biosynthesis in *Malus* crabapple have been well characterized, such as the early biosynthetic genes chalcone synthase (*CHS*; Tai et al., 2014), chalcone isomerase (*CHI*; Geng et al., 2010), flavonoid 3-hydroxylase (*F3^′^H*; Shen et al., 2012), and the late biosynthetic genes dihydroflavonol reductase (*DFR*; Wen et al., 2010), anthocyanidin synthase (*ANS*; Zhang et al., 2015), and UDP-flavonoid glucosyl transferase (*UFGT*; Han et al., 2014). The expression patterns and transgenic expression data have shown that these anthocyanin biosynthetic genes are central to leaf coloration in crabapple.

The regulation of anthocyanin biosynthesis has been associated with MYB family transcription factors (TFs) which influence the expression of flavonoid biosynthetic genes (Aharoni et al., 2001). In apple, three allelic genes, *MdMYBA*, *MdMYB1*, and *MdMYB10* encode regulatory proteins involved in anthocyanin production and fruit coloration, and up-regulation of their expression can promote anthocyanin accumulation (Takos et al., 2006; Ban et al., 2007; Espley et al., 2007). The *MdMYB10* paralog, *MdMYB110a*, has recently been characterized, and increased expression of *MdMYB110a* gene will lead to anthocyanin accumulation in apple flesh (Chagné et al., 2013). *McMYB10* TF, which is identified in leaves and petals of crabapple, and that play a functional role in promoting anthocyanin accumulation by regulating the expression of *McF3’H* and several other structural genes in the anthocyanin biosynthesis pathway (Jiang et al., 2014; Tian et al., 2015). The ubiquitin E3 ligase, MdCOP1, interacts with MdMYB1 and regulates apple fruit coloration by the specific ubiquitination and degradation of MdMYB1 via the proteasome pathway (Li et al., 2012). This finding endows new view into the mechanism of epigenetic modification in regulation of anthocyanin biosynthesis (Li et al., 2012); however, little is known about the transcriptional relationship between *MYB10* and *COP1*.

The commercial value of many ornamental plant species depends on the leaf color (Zhang et al., 2015). *Malus* crabapple has one of the most economically important germplasm for ornamental apple, and this resource also provides a valuable research material for investigating the biosynthesis pathway and molecular basis of diverse plant pigmentation in leaves, flowers and fruits (Tian et al., 2011). Furthermore, the abundance of flavonoids in the leaves and fruits provides an excellent antioxidants source, which can be used as food nutrition additives (Martin, 2013). It is significant for both breeding and genetic engineering of ornamental plants to study the pigmentation mechanisms in crabapple leaves.

In our research, we investigated the expression relationship between the key anthocyanin regulator, *McMYB10*, and its specific ubiquitin E3 ligases McCOP1-1 and McCOP1-2 during leaf development in different crabapple cultivars. Our finding from this study will help future works to promote anthocyanin accumulation in plants and further understand the upstream anthocyanin post-transcriptional regulation mechanisms.

## Materials and Methods

### Plant Material and Growth Conditions Aim

**FIGURE 1:**
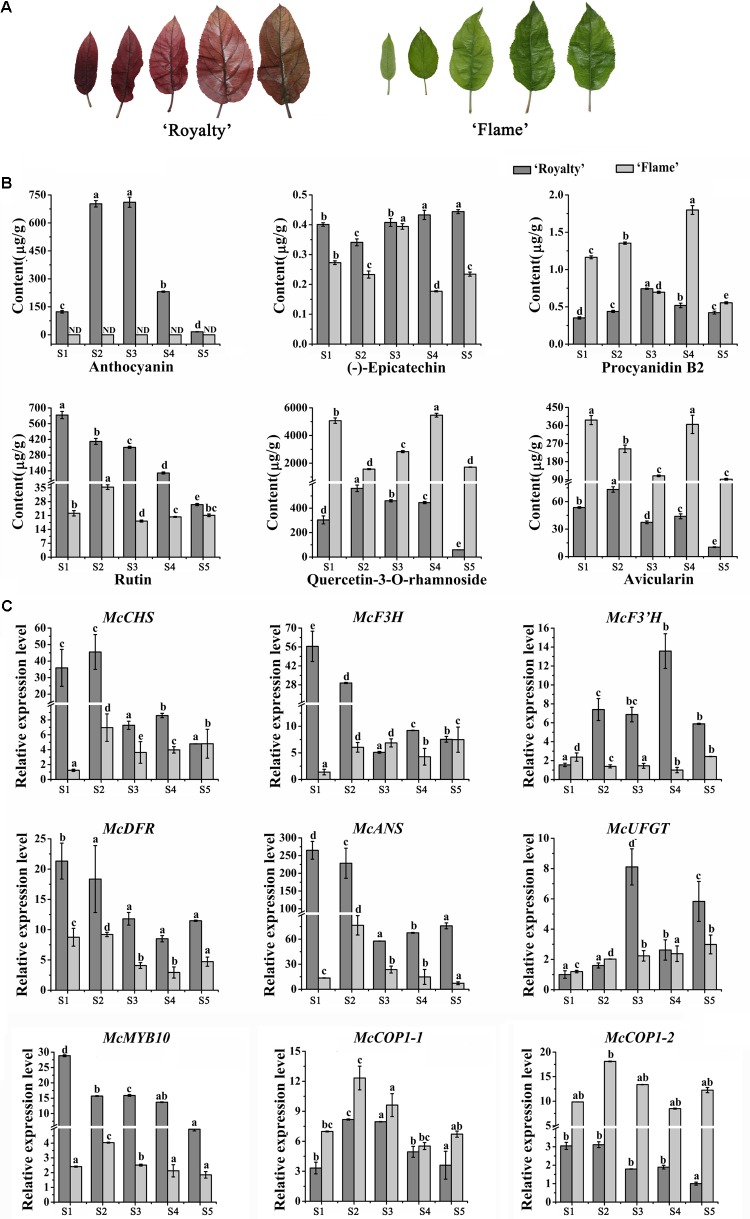
Analysis of flavonoid contents and expression level of anthocyanin biosynthesis-related genes during five leaf developmental stages in the ever-red crabapple cultivar ‘Royalty’ and ever-green crabapple cultivar ‘Flame’. **(A)** The phenotypes of leaves in five leaf developmental stages. **(B)** Flavonoid contents in five leaf developmental stages of ‘Royalty’ and ‘Flame.’ **(C)** The expression patterns of *McCHS*, *McF3H*, *McF3’H*, *McDFR*, *McANS*, *McUFGT*, *McMYB10*, *McCOP1-1*, and *McCOP1-2* were analyzed by qRT-PCR in the leaves of ‘Royalty’ and ‘Flame.’ *Malus 18 S* (DQ341382) as the reference gene. S1 to S5 represents 3 days after budding, 9 days after budding, 15 days after budding, 21 days after budding, 30 days after budding. Error bars indicate the standard error of the mean ± SE of three replicate measurements. The expression levels of flavonoid-related genes were calculated using CFX-Manager-3-1 software (Bio-Rad). Scale bars = 1 cm. Different letters above the bars indicate significantly different values (*P* < 0.05) calculated using one-way analysis of variance (ANOVA) followed by a Tukey’s multiple range test.

The two *Malus* crabapple cultivars were used: (1) an ever-red leaf cultivar, *Malus* cv. ‘Royalty’; (2) an ever-green leaf cultivar, *Malus* cv. ‘Flame’. The 6-year-old cultivars grafted on *M. hupehensis* were planted in the Crabapple Germplasm Resources Nursery (40° 13^′^ N, 116° 46^′^ E, with continental monsoon climate, annual average temperature of 11.2°C and an annual average rainfall of (approximately 610 mm) (Tian et al., 2015). 10–20 annual shoots growing toward the south were selected and 10 leaves collected from the tip to the base of each shoot on May 10th, 2016, when the annual shoots had stopped growing. We chose stages 1 (3 days after budding), 2 (9 days after budding), 3 (15 days after budding), 4 (21 days after budding), and 5 (30 days after budding) of five leaf developmental stages from these two *Malus* crabapple cultivars (**Figure 1A**). The leaf weight at stage 1 was 0.1–0.2 g and at stage 5 was 0.8–1.0 g, and the widths of the leaves collected at each stage were similar to each other. All samples were frozen immediately in liquid nitrogen and stored at -80°C until use (Tai et al., 2014).

The explants of ‘Flame’ and ‘Royalty,’ from 1-year old branches before spring bud germination, were cultured on Murashige and Skoog medium (Murashige and Skoog, 1962) supplemented with 0.1 mg/L 6-benzylaminopurine (6-BA) and 2 mg/L (2,4-dichlorophenoxy) acetic acid (2,4-D) at 23°C with a 12 h light (200 μmol s^-1^ m^-2^)/12 h dark period (Tian et al., 2015). The light in the culture chamber was turned on at 8:00 and turned off at 20:00. Leaves of ‘Royalty’ were collected at eight time points (8:00, 11:00, 14:00, 17:00, 20:00, 23:00, 2:00, 5:00) for the expression assay over the course of a day.

For the apple studies, bagged fruits from adult trees of ‘Red Fuji’ (*M. domestica* ‘Red Fuji’) were harvested 145 days after full bloom (DAFB). Fruits skin was peeled, together with less than 1 mm of cortical tissue, for anthocyanin measurements and other analyses (Xie et al., 2012).

*Nicotiana benthamiana* were grown in a greenhouse at 23°C under 16-h-light/8-h-dark illumination, which was used for BiFC (bimolecular fluorescence complementation, BiFC) experiments.

### Cloning and Sequence Analysis of the Promoter Region of the *McCOP1* Genes

Genomic DNA was isolated from *Malus* cv. ‘Royalty’ and *Malus* cv. ‘Flame’ to isolate the upstream promoter region by using the Plant Genomic DNA Kit (TIANGEN BIOTECH CO., LTD., Beijing, China). Homology-based cloning method were conducted to isolate the regions of upstream DNA immediately adjacent to the transcription start site from the genomic DNA of ‘Royalty’ and ‘Flame’. According to homologous genomic sequences of *Malus* ×*domestica* (ACYM01095562.1) retrieved from the NCBI database *McCOP1* promoter primers were designed as shown in Supplementary Table S1. PLANTCARE database^1^ were used to analyze the *cis*-elements.

### Environmental Condition Treatments

For light treatment, 20-day-old ‘Flame’ seedlings were grown at 23°C for 12h- and 24-h light periods. After 3 days treatment, all samples were collected and frozen in liquid nitrogen and stored at -80°C.

### Construction of Virus Induced Gene Silencing (VIGS) Vectors and *Agrobacterium*-Mediated Infiltration of Crabapple Plants and Apple Fruits

The coding sequence of *McMYB10* (GenBank: JX162681.1) has been deposited in the NCBI (National Center for Biotechnology Information) database previously. Fragments for the pTRV2-GFP-*McMYB10* (342 bp) construct were amplified by gene-specific primers using Taq DNA polymerase (TaKaRa, Ohtsu, Japan) according to the manufacturer’s instructions. The PCR primers are listed in Supplementary Table S1. VIGS vectors carrying the target gene fragments, and pTRV1 and pTRV2-GFP (Tian et al., 2014, 2015), were transformed into *Agrobacterium tumefaciens* strain GV3101 competent cells via a freeze-thaw method (Weigel and Glazebrook, 2006) and selected on kanamycin- rifampicin-containing (50 mg/L) Luria Bertani media plates. Positive clones were verified by sequencing the vector-insert junctions. The harvested bacterial cells were then resuspended to an OD_600_ of 1.0 in infiltration medium (10 mM 2-morpholinoethanesulfonic acid [MES], 200 mM acetosyringone, and 10 mM MgCl_2_) and incubated at room temperature for 3 h. Before infiltration, bacteria carrying pTRV1 and pTRV2-GFP were mixed in a 1: 1 volume ratio (Tian et al., 2014).

For vacuum infiltration, whole crabapple plants and apple fruits were submerged in an *Agrobacterium* suspension and subjected to a vacuum (-25 kPa). The vacuum treatment time and methods were conducted as previously (Yan et al., 2012). The infiltrated crabapple plants were rinsed with sterile water and cultured on MS medium. Fifteen plants from ‘Royalty’ and fifteen ‘Red Fuji’ apple fruits were used for *McMYB10* expression silencing studies. Infiltrated plants and fruits were then cultured in the dark for 12–16 h and transferred to environmentally controlled growth chambers.

### RNA Extraction

Tissue samples from areas reflecting the silencing and overexpression phenotypes were collected to analyze the effects of target gene and anthocyanin biosynthesis genes expression. Tissues infected by *Agrobacterium* carrying vectors with no host gene fragment insert, or from non-infected plants were selected as control. Three independent biological replicates were analyzed. RNA plant plus reagent (TIANGEN BIOTECH CO., LTD., Beijing, China) were used to extracted total RNA from crabapple leaves and apple fruits. DNase (TIANGEN BIOTECH CO., LTD., Beijing, China) treatment was performed according to the manufacturer’s instructions. Total RNA was synthesized to first-strand cDNA by the Reverse Transcriptase M-MLV (RNase H^-^) kit (TaKaRa, Ohtsu, Japan).

### Quantitative RT-PCR Analysis (qRT-PCR)

The qRT-PCR experiment was performed using the SYBR^®^ Premix Ex TaqTM II (Perfect Real Time) (TaKaRa, Ohtsu, Japan) and the CFX96TM Real Time System (Bio-Rad, Hercules, CA, United States). The PCR amplification conditions were as previously described (Tian et al., 2015), and transcript levels were determined by relative quantification using the *Malus* 18S ribosomal RNA gene (DQ341382) as the internal control and the 2^-ΔΔC_T_^ analysis method (Livak and Schmittgen, 2001). Specific primers (Supplementary Table S1) for qRT-PCR analysis were designed using the primer 5 software^2^.

### High Pressure Liquid Chromatography (HPLC) Analysis

Crabapple leaf samples [approximately 0.8–1.0 g fresh weight (FW)] and apple fruit samples (approximately 0.5–0.3 g FW) were subjected to extraction with 10 mL extraction solution (methanol: water: formic acid: trifluoroacetic acid = 70: 27: 2: 1) at 4°C in the dark for 72 h, shaking every 6 h (Tian et al., 2015). The detection methods were as previously described (Revilla and Ryan, 2000). All samples were analyzed in three biological triplicates (extracted from three different pools of leaves).

### Yeast One-Hybrid (Y1H) Assay

The Y1H screening was performed in terms of the Matchmaker Gold Yeast One-Hybrid Library Screening System (Clontech, Ohtsu, Japan), as recommended by the manufacturer. The assay used the yeast strain Y1HGold, which is unable to grow in the absence of uracil. The Sp1 (CCCGCC) and MBS (CAGTTA) *cis*-elements, which are potential MYB10 binding sites in the *McCOP1* promoters, were identified using the PLANTCARE database^1^. A trimer of Sp1 and a trimer of MBS were synthesized and the double trimer mutant sequence were cloned in front of the minimal promoter of the pAbAi vector to give the bait vectors (the synthesized double trimer sequence and mutant double trimer sequence are shown in Supplementary Table S2). The pMBS-box&Sp1-box-AbAi vector and pMutant-AbAi vector were transformed into Y1HGold cells. The *McMYB10* sequence was cloned into the pGADT7-Rec vector to give the prey vector and transformed into the Y1HGold strain containing the pMBS-box&Sp1-box-AbAi vector or pMutant-AbAi vector. Transformed cells were plated on SD/- Leu and SD/- Leu/+AbA to select for colonies whose prey proteins had activated the AbAr reporter. Positive colonies were analyzed by yeast colony PCR, and PCR products were sequenced. Yeast transformed with p53-AbAi or pMBS-box&Sp1-box-AbAi were used as negative control, and the yeast transformed with p53-AbAi and pGADT7-Rec-p53 was used as positive controls.

### Electrophoretic Mobility Shift Assay (EMSA)

The pMAL-C2X expression vector containing the open reading frame sequence of *McMYB10* (New England Biolabs, Ipswich, MA, United States) were transformed into Rosetta (DE3) *Escherichia coli* competent cells. To facilitate purification of recombinant proteins, the maltose binding protein (MBP)-tag encoded in the pMAL-C2X vector was used. To induce McMYB10 protein accumulation, isopropyl β-D-1-thiogalactopyranoside (IPTG) (0.3 mM) was added to the cultures at 170 rpm for 6 h at 28°C (Tian et al., 2015). One-Stop MBP-Tagged Protein Miniprep Pack (BioLab Co. Ltd., Beijing, China) was used to purify the recombinant protein. EMSA reactions were prepared according to the manufacturer’s protocol (LightShif^®^ Chemiluminescent EMSA Kit; Thermo Fisher Scientific, Waltham, MA, United States). The oligonucleotides used for EMSA are listed in Supplementary Table S3. For each EMSA reaction, approximately 10 μg of purified McMYB10 recombinant protein was used (Tian et al., 2015).

### Yeast Two Hybrid (Y2H) Assay

Yeast transformants were screened on SD media (-Leu/-Trp/-His/-Ade) according to the manufacturer’s instructions (Clontech, Palo Alto, CA, United States). To generate an fusion with the GAL4 activation domain, the McCOP1 coding region was cloned into pGBKT7 vector. McMYB10 coding sequence was cloned in pGADT7 to generate an fusion which contains the GAL4 DNA-binding domain (GBD). By lithium acetate method, two plasmids which constructed above were co-transformed into the yeast AH109 strain and cultured at 28°C. The resultant yeast transformants were screened on SD (-Leu/-Trp/-His/-Ade) medium according to the manufacturer’s instructions (Clontech, Mountain View, CA, United States) (Li et al., 2012). The positive transformants were transferred to medium (-Leu/-Trp/-His/-Ade) and containing *X*-gal (5-bromo-4-chloro-3-indolyl-β-D-galactopyranoside acid) at 28°C for 2 days, which selected by the medium lacking Trp and Leu (-Trp/-Leu) (Li et al., 2012).

### Bimolecular Fluorescence Complementation (BiFC) Assay

Bimolecular fluorescence complementation assay was performed as described (Chen et al., 2013). Full-length sequences of McMYB10 and McCOP1 were cloned into pSPYCE-35S/pUC-SPYCE and pSPYNE-35S/pUC-SPYNE vectors, respectively. Negative controls were used as the pairs of the above fusion protein with corresponding empty vector. These vectors were transformed into the *Agrobacterium* strain GV3101. *Agrobacterium* cells were resuspended in infiltration medium (10 mM MgCl_2_, 10 mM MES, and 100 mM acetosyringone) and incubation for 2 h at room temperature. The suspension was infiltrated into leaves as different combination as mentioned above into tobacco leaves (*N. benthamiana*). After 72 h, the GFP fluorescence were captured by a confocal laser-scanning microscope (FV1000, Olympus, Tokyo, Japan) (Chen et al., 2013).

## Results

### Expression Level of Anthocyanin-Related Genes and Determination of Anthocyanin Content During Leaf Development

Based on their extreme leaf color phenotypes, *Malus* crabapple cultivars, *Malus* cv. ‘Royalty’ and *Malus* cv. ‘Flame’ were chosen for color analysis (**Figure 1A**). The abundance of anthocyanins and flavonoid compound composition in the leaves of ‘Royalty’ and ‘Flame’ at 5 development stages were evaluated by HPLC (**Figure 1B**).

A gradual decreased accumulation trend in anthocyanin content was found in ‘Royalty’ leaves, and there is no obviously accumulation of anthocyanin in ‘Flame’ by HPLC analysis. The concentration of flavonols (quercetin-3-*O*-rhamnoside and rutin) and avicularin gradually decreased in ‘Royalty’ and ‘Flame’ leaves during leaf development, with the exception of quercetin-3-*O*-rhamnoside and avicularin in stage 4 of ‘Flame’. The abundance of (-)-epicatechin increased during the development of ‘Royalty’ leaves, and procyanidin B2 reached its maximum in the third stage where after levels decreased. (-)-epicatechin and procyanidin B2 in ‘Flame’ showed completely opposite trends (**Figure 1B**).

Previous studies have shown that MdCOP1 can specifically ubiquitinate and degrade MdMYB1 via the proteasome pathway (Li et al., 2012). However, the expression patterns of *COP1* and the expression relationship between *MYB10* and *COP1* is still unknown. To assure that the McMYB10 as a target of McCOP1, yeast two hybrid and BiFC assay were conduct and the results showed that McMYB10 can interact with McCOP1-1 and McCOP1-2, respectively (Supplementary Figure S1). To further confirm the relationship between the expression patterns of *McCOP1* and anthocyanin accumulation, we compared the expression profiles of *McCOP1* genes (*McCOP1-1* and *McCOP1-2*, MDP0000245133 and MDP0000259614), *McMYB10* and different anthocyanin biosynthetic genes between the ever-red leaved ‘Royalty’ and ever-green leaved ‘Flame’ cultivar during different leaf developmental stages by qRT-PCR. The expression levels of the anthocyanin biosynthetic genes were higher at the early stages and then decreased at the late development stages in ‘Royalty’ except *McF3’H* and *McUFGT*, which showed increase expression almost through the entire leaf development (**Figure 1C**). In ‘Flame’ the expression of *McCHS*, *McF3H*, *McF3’H*, and *McUFGT* gradually increased with leaf development, while *McDFR* and *McANS* exhibited opposite expression characteristics. The transcript levels of *McMYB10* was found to be positively correlated with the expression of the anthocyanin biosynthetic genes, and also positively correlated with the anthocyanin content in ‘Royalty’ and ‘Flame’ leaves (**Figure 1C**). We also noted that the transcript levels of the two *McCOP1* genes, especially *McCOP1-2*, showed as similar pattern of expression with *McMYB10* (**Figure 1C** and Supplementary Table S4).

### Expression Level of Anthocyanin-Related Genes and Anthocyanin Content Over the Course of a Day

**FIGURE 2:**
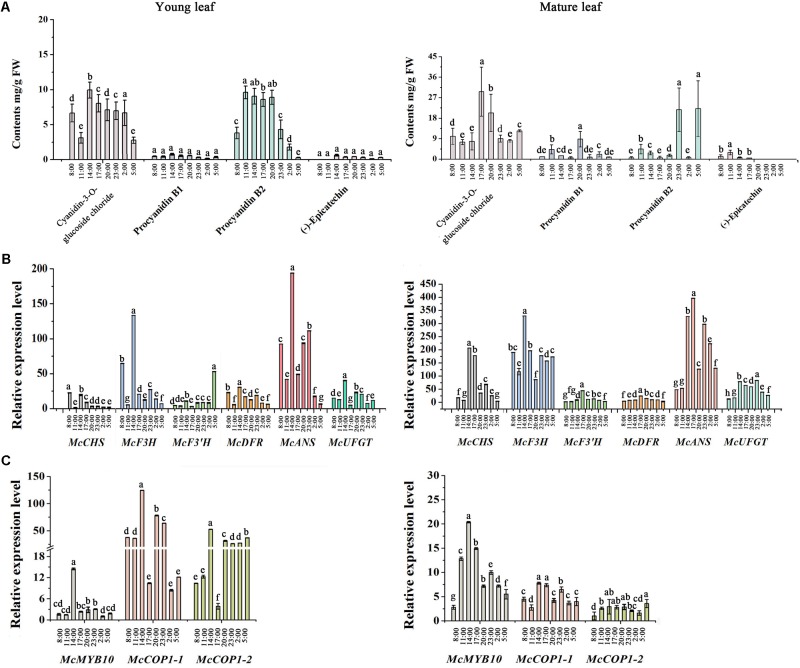
Analysis of flavonoid contents and expression level of anthocyanin biosynthesis-related genes during eight time points from 8:00 am to 5:00 pm in young and mature leaves of the *Malus* crabapple ever-red cultivar ‘Royalty.’ **(A)** The content of the main flavonoid compounds at eight time points in young and mature leaves of ‘Royalty.’ **(B,C)** The expression patterns of *McCHS*, *McF3H*, *McF3’H*, *McDFR*, *McANS*, *McUFGT*, *McMYB10*, *McCOP1-1*, and *McCOP1-2* were analyzed in the young and mature leaves of ‘Royalty’ by qRT-PCR. *Malus 18 S* (DQ341382) was used as the reference gene. Error bars indicate the standard error of the mean ± SE of three replicate measurements. The expression levels of flavonoid-related genes were calculated using CFX-Manager-3-1 software (Bio-Rad). Different letters above the bars indicate significantly different values (*P* < 0.05) calculated using one-way analysis of variance (ANOVA) followed by a Tukey’s multiple range test.

To further assess the potential relationship between *McCOP1* and *McMYB10*, we determined the expression level of anthocyanin-related genes and flavonoid accumulation at eight time points during a day in young leaves and mature leaves in ‘Royalty’. The leaves had no phenotypical variation during the day and the HPLC results revealed that the anthocyanin accumulated during light exposure and decreased when the light was removed, and the highest accumulation appeared at 14:00 in young leaves and 17:00 in mature leaves, respectively (**Figure 2A**). The content of procyanidin B2 have the same variation trend with anthocyanin content, and the content of procyanidin B1 and (-)-epicatechin almost same at eight time points in young leaves. Meanwhile, the contents of procyanidin B1, procyanidin B2 and (-)-epicatechin increased at 11:00 time point and then decreased with the time, except procyanidin B1 at 20:00 and procyanidin B2 at 23:00 and 5:00 time points in mature leaves. Meanwhile, the expression level of *McCOP1-1*, *McCOP1-2*, and *McMYB10* increased during daylight and gradually decreased in the night in both young and mature crabapple leaves (**Figure 2C**). The same expression trends were observed for flavonoid biosynthetic genes (**Figure 2B**). Correlation analysis suggested that *McCOP1-1* have high correlation with *McMYB10* expression in crabapple leaves in the light and dark, but *McCOP1-2* have high correlation with *McMYB10* expression in the young and mature leaves only in light (Supplementary Table S5). Taken together, we hypothesize that there may be some potential connection between the expression of *McCOP1* and *McMYB10*.

### *McMYB10* Activates the *McCOP1* Promoters

**FIGURE 3:**
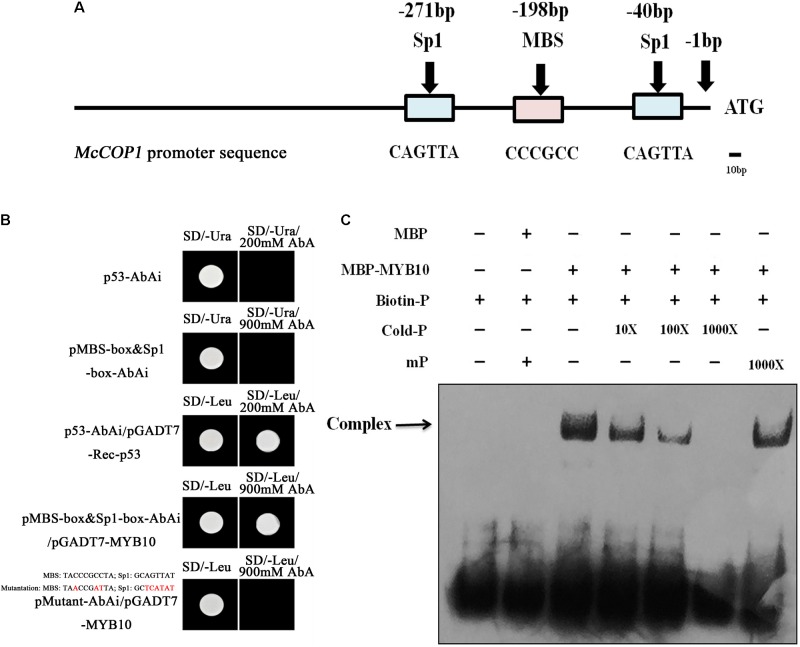
The relationship between McMYB10 and the *McCOP1* genes. **(A)** Schematic overview of MYB binding sites in the *McCOP1s* promoter, with MYBCORE elements located at –236 bp of the *McCOP1* promoters from ATG sites. As there was no significant structural difference between two *McCOP1* promoters, we represented the two promoter sequences as ‘*McCOP1* promoter sequence.’ The line represents the DNA sequences of *McCOP1*, the blue box represents Sp1 *cis*-element and the pink box represents MBS *cis*-element. **(B)** Interaction of the McMYB10 protein with the promoters of the *McCOP1* genes as revealed by yeast one-hybrid assays. The yeast strain Y1HGold, which is unable to grow in the absence of uracil was used for the assay. The core sequence of the *McCOP1* promoters Sp1 and MBS elements were synthesized and cloned into the pAbAi vector as bait, then transformed into the Y1HGold yeast strain. MYB10 was cloned into the prey vector, pGADT7-Rec. To select for colonies which can activate the AbAi reporter, transformed cells were grown on SD/– Leu and SD/– Leu/+AbA medium. Yeast transformed with p53-AbAi or pMBS-box&Sp1-box-AbAi were used as negative control, and the yeast transformed with p53-AbAi and pGADT7-Rec-p53 was used as positive controls. The mutations in MBS and Sp1 box were highlight in red. **(C)** Gel-shift analysis of McMYB10 binding to the promoters of the *McCOP1* genes. Biotin-labeled probe or mutant probe (mP) were incubated McMYB10 purified protein, respectively. Non-labeled probes at 10- to 1,000-fold concentrations and labeled mutant probes (1,000-fold) were added to the experimental for the competition test.

The *McMYB10* and *McCOP1* genes exhibited similar expression patterns during crabapple leaf development. To test the relationship between the *McCOP1* genes and *McMYB10*, a 1,500 bp fragment of the *McCOP1-1* (MG457755) promoter and a 1,123 bp fragment of the *McCOP1-2* (MG457756) promoter were cloned from ‘Royalty’ leaves. The nucleotide sequence identity of two promoters was 96% and there was no significant structural difference between them (Supplementary Figure S2). We also analyzed the *cis*-elements in *McCOP1-1* and *McCOP1-2* promoters using the PLANTCARE database. The results showed that the MBS and Sp1 *cis*-elements, which are known to be important for MYB TF binding, are present -173 and -40 bp upstream of the *McCOP1* ATG start site (**Figure 3A**). We hypothesized that the *McCOP1* genes might be McMYB10 targets. Y1H was employed to characterize the ability of McMYB10 to bind to the *McCOP1* promoters. We generated a sequence containing the trimer of the MBS and sp1 *cis*-elements, and also generated a mutant sequence containing the double mutant trimer. Colonies were plated on SD/-Leu/+AbA to select for activation of the AbAr reporter. This reporter was not activated in pMutant-AbAi/pGADT7-MYB10 or in the controls, p53-AbAi and pMBS-box&Sp1-box-AbAi, it was in the pMBS-box&Sp1-box-AbAi/pGADT7-McMYB10 cells (**Figure 3B**). To further detect whether McMYB10 proteins binds directly to the MYB *cis*-elements in the *McCOP1-1* and *McCOP1-2* promoters, DNA fragments containing the MBS and sp1 *cis*-elements were served as probes to examine possible interactions with McMYB10 protein in an EMSA assay (**Figure 3C**). McMYB10 protein bound to the biotin labeled *proMcCOP1* probe by Gel shift assays. This binding diminished gradually with an increasing concentration of un-labeled probe, while there was no evidence of competition using a mutated probe. The specific interaction between McMYB10 and the *McCOP1* promoters were confirmed by the specificity of competition.

### Light Alters Anthocyanin Accumulation and the Expression of *McMYB10* and *McCOP1*

**FIGURE 4:**
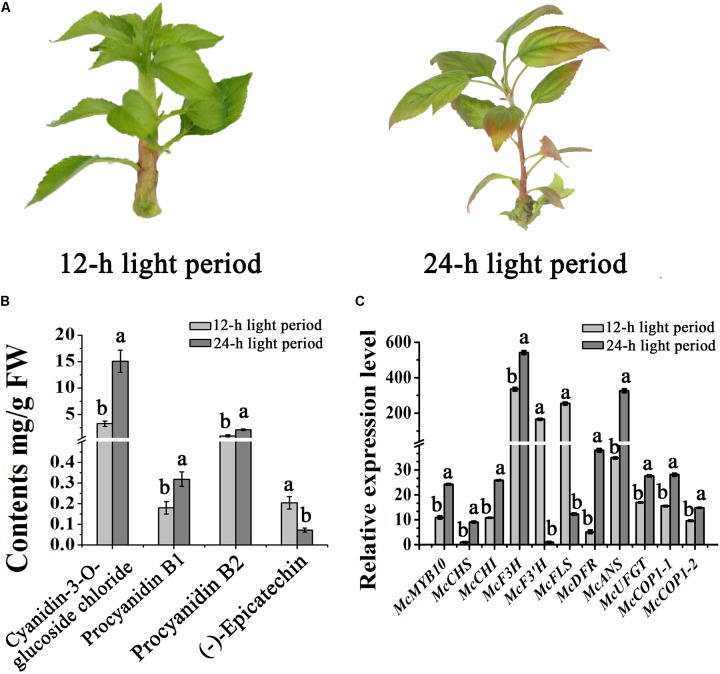
Different light treatments in crabapple foliage. **(A)** ‘Flame’ tissue culture plants under different light treatments. After 3 days treatment, red color appeared in the plants treated with light for a 24-h period (right panel). **(B)** High pressure liquid chromatography (HPLC) analysis showing the variation of flavonoids in crabapple leaves under different light treatments. **(C)** qRT-PCR was conducted to analysis the expression levels of anthocyanin biosynthesis-related genes in ‘Flame’ plantlets. Error bars indicate the standard error of the mean ± SE of three replicate measurements. Different letters above the bars indicate significantly different values (*P* < 0.05) calculated using one-way analysis of variance (ANOVA) followed by a Tukey’s multiple range test.

Light can promote the expression of *MYB10*, and promotes anthocyanin accumulation in apple (*Malus* ×*domestica*) (Takos et al., 2006). To further understand the impact of light on the expression of *McMYB10* and *McCOP1* in crabapple leaves, we exposed 20-day-old ‘Flame’ seedlings to continuous light. After 3 days of treatment, the leaves showed red color in new buds, mature leaves and stems in the 24-h light treated plants, the 12-h treated plants did not. We observed no obvious phenotypic variation between plants grown in the 12-h treatment. HPLC analysis was used to determine the flavonoid content of differently treated plants. The results showed that anthocyanin abundance increased almost threefold in leaves exposed to 24-h light more than 8-h light treated plants (**Figures 4A,B**). To further confirm that the red color phenotypes in 24-h light treatment leaves correlated with increased expression of the *McMYB10*, qRT-PCR was performed to analyze the expression level of *McMYB10* and anthocyanin biosynthetic genes (*McCHS, McCHI, McF3H, McDFR*, *McANS*, and *McUFGT*). The results demonstrated that the expression level of most of anthocyanin biosynthesis genes were significantly increased in the leaves of plants treated for 24-h compared with the 12-h light treatment. However, *McF3’H* and *McFLS* expression was inhibited when long light treatments were employed (**Figure 4C**). We also tested the expression level of *McCOP1-1* and *McCOP1-2* during different light treatments, and found that the transcription of these two genes was significantly higher in leaves treated with light for 24-h than for 12-h. These results suggested that long illumination time promoted anthocyanin accumulation as a result of the increased *McMYB10* expression, and the abundant expression of *McMYB10* was accompanied with an increased expression of the *McCOP1* genes.

### Variation in the Expression of *MYB10* in Crabapple Leaves

**FIGURE 5:**
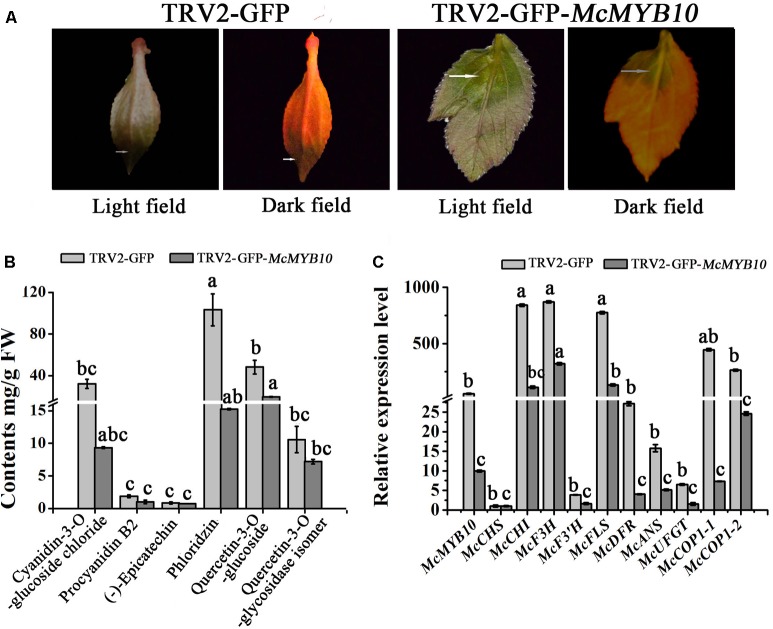
Silencing of *McMYB10* in the ever-red cultivar ‘Royalty’. **(A)** After 14 days infiltration, the faded red leaf phenotype was observed. **(B)** Flavonoid contents in infiltrated ‘Royalty’ plantlets in μg/g fresh weight (FW). **(C)** qRT-PCR was conducted to analysis the expression levels of anthocyanin biosynthesis-related genes. Error bars indicate the standard error of the mean ± SE of three replicate measurements. Different letters above the bars indicate significantly different values (*P* < 0.05) calculated using one-way analysis of variance (ANOVA) followed by a Tukey’s multiple range test.

In order to establish the regulatory relationship between *McMYB10* and *McCOP1*, we silenced *McMYB10* gene in ‘Royalty’ leaves by VIGS using the TRV-GFP vector. The viral plasmid pTRV2-GFP-*McMYB10* comprising a partial *McMYB10* open reading frame (ORF) was used to infiltrate crabapple leaves, and pTRV2-GFP vector was used as a control. Two weeks after infection, faded-purple phenotype was observed in new leaves infiltrated with the pTRV2-GFP-*McMYB10* vector, and the control leaves accumulated anthocyanins and developed red coloration in new leaves (**Figure 5A**).

The results of anthocyanin content confirmed that the silenced leaves contained less anthocyanin than the control. The cyanidin concentration in the TRV2-GFP-*McMYB10* infiltrated leaves (3 μg/g FW) was much lower than in the TRV2 infiltrated leaves (38 μg/g FW). HPLC analysis also showed a decrease in the concentration of phlorizin and quercetin-3-*O*-glucoside in the *McMYB10*-silenced lines (**Figure 5B**). qRT-PCR revealed that the transcription of endogenous *McMYB10* gene was greatly decreased in TRV-GFP-*McMYB10* infiltrated leaves, as were the transcript levels of the anthocyanin biosynthetic genes, *McCHS*, *McF3H*, *McANS*, and *McUFGT* (**Figure 5C**). Down-regulation of the expression of *McMYB10* also affected the transcription of the *McCOP1* genes in silenced leaves, and a lower expression level of the *McCOP1* genes was also observed in TRV2-GFP-*McMYB10* infiltrated leaves compared with control leaves.

### Altered Expression of *McMYB10* in Apple Fruits

**FIGURE 6:**
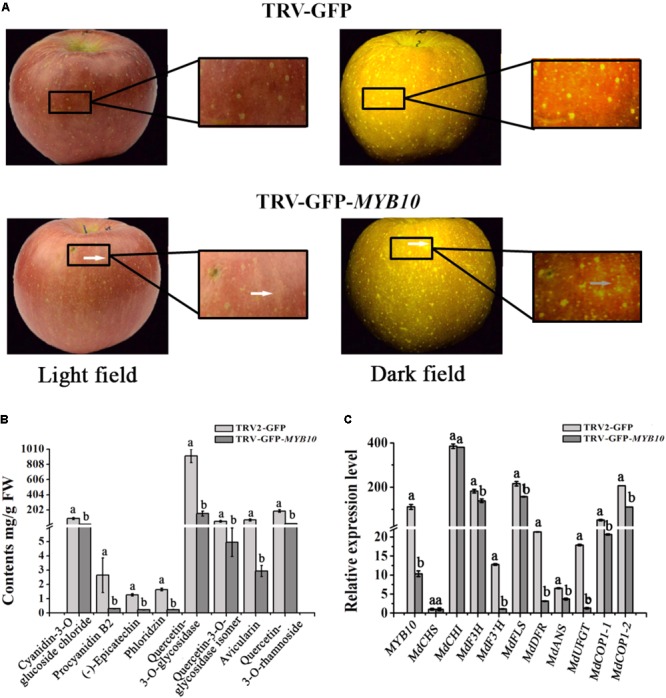
Transiently silencing of *MYB10* in apple fruit. *MYB10* was silenced using the TRV-GFP-*MYB10* vector. Apple fruit vacuum infiltration with the empty TRV vectors were used as controls. **(A)** Apple fruit peel coloration around the infiltrated sites of suppressed (TRV-GFP-*MYB10*) apples. **(B)** Flavonoid compound levels around the infiltrated sites of apple peels were analyzed by HPLC in μg/g FW. **(C)** qRT-PCR was conducted to analyze the expression levels around the infiltrated sites, the expression level of *MYB10* represent the transcription level of endogenous *MdMYB10* in silenced fruits. Error bars indicate the standard error of the mean ± SE of three replicate measurements. Different letters above the bars indicate significantly different values (*P* < 0.05) calculated using one-way analysis of variance (ANOVA) followed by a Tukey’s multiple range test.

McMYB10 has an identical predicted amino acid sequence to apple MdMYB10 (Tian et al., 2015). To confirm the hypothesis that *McMYB10* can regulate the transcription of *McCOP1*, we infiltrated pTRV2-GFP-*MYB10* into apple fruits. Silencing of *MYB10* repressed fruit coloration, with the infiltrated sites having a yellow or white striped appearance in pTRV2-GFP-*MYB10* infiltrated ‘Red Fuji’ fruits (**Figure 6A**). HPLC analysis showed that silencing of *MYB10* generated a significantly declined in anthocyanin contents and several other flavonoid components, such as PAs (Proanthocyanidin) [(-)-epicatechin and procyanidin B2], flavonols (quercetin-3-*O*-glycosidase, quercetin-3-*O*-glycosidase isomer and rhamnoside), phlorizin and avicularin (**Figure 6B**). The expression levels of the *MdMYB10* gene and several flavonoid biosynthetic genes in the transformed apple fruit were evaluated using qRT-PCR. We observed that changes in the anthocyanin content was accompanied by reduced transcript levels of *MYB10*. Around the infiltration sites, compared with the control, the expression of *MdCHS*, *MdDFR*, *MdANS*, *MdF3’H*, and *MdUFGT* were significantly down-regulated, while the expression of *MdCHI*, *MdF3H*, *MdFLS* showed no significant variation (**Figure 6C**). Furthermore, the variation in expression of *MYB10* also affected the expression of the *COP1* genes, so the lower expression of *MYB10* in the *MYB10*-silenced fruit peels resulted in a lower expression of these. We deduced that *MYB10* may modulated the expression of *COP1* to regulate its own transcript levels.

## Discussion

In apple, *MYB10*, *MYB1*, and *MYBA*, which maps to the same locus, have already been independently characterized, and functional assays have revealed that these TFs are pivotal regulators of anthocyanin accumulation and fruit peel pigmentation by *trans*-activating anthocyanin biosynthetic genes (Takos et al., 2006; Ban et al., 2007; Espley et al., 2007). A recent study showed that *MYB110a*, a paralog of *MYB10*, is correlated with a red-flesh phenotype in apple, and that *MYB10* and *MYB110a* have conserved functions, but have significant expression variation during fruit maturity in some cultivars (Chagné et al., 2013).

Plants have several self-regulation mechanisms to adapt to environmental variation and different developmental stages, which help avoid excessive accumulation of certain compounds (Brand et al., 2000; Schoof et al., 2000; Dai et al., 2007; Ryan et al., 2017). DELLA proteins act suppressors of gibberellic acid signaling pathway and are immediately degraded in GA presence. Interestingly, DELLA proteins regulate many target genes, including GA-biosynthetic enzyme GA 20-oxidase in *Arabidopsis*. Therefore, the DELLA-GAF1 (GAI-ASSOCIATED FACTOR1, a DELLA interactor) complex is a main component in controlling endogenous GA levels and GA signaling by regulating the expression of GA biosynthetic genes (Fukazawa et al., 2017). EPIDERMAL PATTERNING FACTOR1 (EPF1) and its primary receptor ERECTA-LIKE1 (ERL1) specify the proliferation-to-differentiation switch within the stomatal cell lineages by regulating MUTE expression. Meanwhile, MUTE can induce the expression of EPF1. This self-inhibition mechanism ensures appropriate stomatal development (Qi et al., 2017). Ubiquitination is an enzymatic post-translational modification that can regulate the protein balance. In apple and *A. thaliana*, anthocyanin-correlated MYB TFs are regulated at the pos-translational level, such as the *COP1* genes, which physically interact with *MYB1* (*PAP1*) and conduct its ubiquitination and degradation to control the coloration of fruit peel and leaves (Li et al., 2012; Maier et al., 2013). However, it remains unknown how the *COP1* genes are activated and ubiquitinate *MYB10* to coordinate plant coloration.

In our study, we found that the transcription level of *McMYB10* has a positive correlation with the transcription of *McCOP1* and anthocyanin accumulation during leaf development in a red-colored leaf crabapple cultivar. We deduced that there is a relationship between *McMYB10* and *McCOP1* at the transcriptional level. We therefore cloned the *McCOP1-1* and *McCOP1-2* promoters and detected several MYB TF binding sites, consisting of the MYBCORE *cis*-element. DNA-binding assays demonstrated that the *McMYB10* TF binds specifically to the promoter of these *McCOP1* genes. Furthermore, light-induced McMYB10 expression and transient genetic transformation also proved that *McMYB10* expression control the transcription of the *McCOP1* genes. From these results, we propose that the *McMYB10* TF can induce the expression of *McCOP1* and modulate its own expression by ubiquitination of the McMYB10 protein.

Meanwhile, we also noticed that the expression level of *McCOP1* genes was still relative high in the night, even though the lower expression level of *McMYB10* in that time point. And there is no significantly expression relationship between *McMYB10* and *McCOP1-2* in the night in both young and mature leaves. We deduced that some other TFs may be involved in activating the expression of *McCOP1* under dark condition. The unknown TF may work together with McMYB10 to regulate the expression of *McCOP1* in the light and dark, respectively, to guarantee there are enough McCOP1 to conduct McMYB10 protein ubiquitination. In addition, McCOP1-1 and McCOP1-2 may have different function mechanism in dark. We will focus to the relate regulation mechanism in our future work.

**FIGURE 7:**
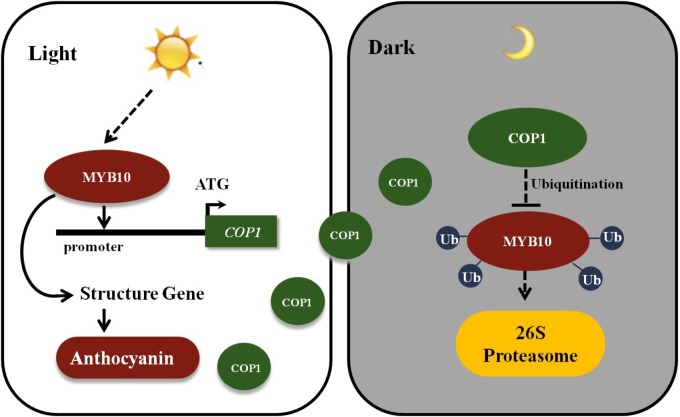
Schematic showing MYB10 modulates the expression of *COP1* to regulate its own expression. MYB10 activates the transcription of the anthocyanin biosynthesis genes and induces the anthocyanin accumulation in crabapple leaves in the light. Meanwhile, MYB10 also promotes the expression of *COP1* genes by binding to their promoter. To avoid excessive accumulation of MYB10, E3 ubiquitin ligase COP1 proteins interact with MYB10 and transferred MYB10 into 26S proteasome to conduct its ubiquitination and degradation in the dark. Ub, Ubiquitin.

The MYB TFs were mainly focused on their functions in previous researches in flavonoid biosynthesis, and the research by Li et al. (2012) showing that *MdCOP1* ubiquitinate *MdMYB1* provide us with new insights into post-translational modification in the plant coloration process. In *Malus* plants, fruits, and leaves showed a rapid response to light in red coloration by an elevation in the expression of the *MYB10* gene. A 23-bp repeat in the promoter sequence of MYB10 alleles was found to be present only in red-fleshed and leaves *Malus* plants (Espley et al., 2009; Tian et al., 2015). Moreover, this promoter repeat sequence was responsible for the accumulation of anthocyanins, and with the increased of repeat units will results in an increase in self-transactivation by the MYB10 protein (Espley et al., 2009). However, plants will not continuously accumulate anthocyanins due to *COP1-*meditated ubiquitination. Furthermore, due to the higher expression of *MYB10* and the *COP1* genes in the daylight and their lower expression in the night, we deduce that *MYB10* not only promotes its own the expression by binding the R6 promoter (six 23-bp sequence repeats), but also activates the expression of the *COP1* genes in the light and ubiquitination of the *MYB10* protein in the dark, to avoid excessive expression of *MYB10* (**Figure 7**). This research reveals novel biotechnological strategies to modulate anthocyanin biosynthesis by genetically modify methods in ornamental plants.

## Author Contributions

JT and Y-CY conceived and designed the experiments. K-TL, M-CC, and Y-HK performed the experiments. JZ, K-TL, T-TS, and HG analyzed the data. JT, Y-CY, and JZ contributed reagents, materials, and analysis tools. JT, K-TL, and M-CC wrote the paper.

## Conflict of Interest Statement

The authors declare that the research was conducted in the absence of any commercial or financial relationships that could be construed as a potential conflict of interest.The reviewer SM and handling Editor declared their shared affiliation.

## Footnotes

1 http://bioinformatics.psb.ugent.be/webtools/plantcare/html/

2 https://www.bioprocessonline.com/doc/primer-premier-5-design-program-0001
