# Supplementary material for: McMYB10 Modulates the Expression of a Ubiquitin Ligase, McCOP1 During Leaf Coloration in Crabapple
Source: Front Plant Sci. 2018 Jun 4;9:704. doi: 10.3389/fpls.2018.00704 (PMC5994411; doi:10.3389/fpls.2018.00704)
Supplement: Supplementary file 1 [file Table_1.DOCX]

**Supplementary Table S1.** Primer sequences used in this study.

| **Accession**  **number** | **ID** | **Sequence (5’-3’)** | **Used for** |
| --- | --- | --- | --- |
|  |  |  |  |
|  | pBI121-McMYB10-F | GGATCCATGGAGGGATATAACGAA | pBI121-*McMYB10* generation |
|  | pBI121-McMYB10-R | CCCGGGTTCTTCTTTTGAATGATT |  |
|  | pTRV-McMYB10-F | GGATCCATGGAGGGATATAACGAA | TRV2-*McMYB10* generation |
|  | pTRV-McMYB10-R | CCCGGGTTTCACCGTTTTCATGCG |  |
|  | PMcMYBCOP1-F | GAAGTATTCGGATTTCACGGTGGGGAC | McCOP1 promoter cloning |
|  | PMcMYBCOP1-R | GACCTCCCAATTCTCGAAGGCCAAA |  |
|  | G-McMYB12b-R | GCTCTAGATTTGGTATTGAGGAGGATGG |  |
| KJ020112 | McMYB10-F | CAGCAAGTGCTAAGATGCAAAC | qRT-PCR |
|  | McMYB10-R | GCTATCAAAGACCACCGATTG |  |
| KJ020111 | McMYBCOP1-F | AGCAGCAAGTGCTACGATGAC | qRT-PCR |
|  | McMYBCOP1-R | GCTATCAAAGACCACCGATTG |  |
| FJ599763 | McCHS-F | TGACCGTCGAAGTTCGC | qRT-PCR |
|  | McCHS-R | TTTGTCACACATGCGCTGGA |  |
| FJ817485 | McCHI-F | AGGAGTTGTCGGAGTCCGTT | qRT-PCR |
|  | McCHI-R | ACTTTCTCAGAGTATTGCTGGCC |  |
| FJ817486 | McF3H-F | ACGAAGACGAGCGTCCAAAG | qRT-PCR |
|  | McF3H-R | CTCCTCCGATGGCAAAGCAA |  |
| KF481684 | McF3’H-F | CGTTGCTGTCGCTCACGGATGA | qRT-PCR |
|  | McF3’H-R | ATGACGTGTCAGTGCCAGCTGTG |  |
| FJ817487 | McDFR-F | CCGAGTCCGAATCCGTTTGT | qRT-PCR |
|  | McDFR-R | CCTTCTTCTGATTCGTGGGGT |  |
| FJ817488 | McANS-F | CACAGGGGCATGGTGAACAA | qRT-PCR |
|  | McANS-R | TTCACTTGGGGAGCAAAGCC |  |
| KF495603 | McUFGT-F | TGGGCGGACACCAATCA | qRT-PCR |
|  | McUFGT-R | ATGTCTCCACCGCACCA |  |
| KF495602 | McFLS-F | ACGAGCAACCGGGAATCACAACTG | qRT-PCR |
|  | McFLS-R | CCCAGTTGGAGCTGGCCTCAGTA |  |
| KT276930 | McANR1-F | AACCACAAGAAGGTCTCCCAC | qRT-PCR |
|  | McANR1-R | CCCTTGGATTGCTGGTTTGAT |  |
| KT276931 | McANR2-F | ACCCCTGTCAACTTTGCCTCA | qRT-PCR |
|  | McANR2-R | CCAAACCTGTTCCCTCAAGTGTAT |  |
| KT276929 | McLAR1-F | TTTATCAAAGGATGCCAGGTT | qRT-PCR |
|  | McLAR1-R | CATCCAAGGTCCTGAAAGAAT |  |
| KT276928 | McLAR2-F | TGGAGAAACGCAAGGTTAGAC | qRT-PCR |
|  | McLAR2-R | TCACCATAGATTTGGAACCGA |  |
| JX162681 | McMYB10-F | ACGCCACCACAAACGTCGTCG | qRT-PCR |
|  | McMYB10-R | GGCGCATGATCTTGGCGACAGT |  |
| ACQ45201 | MdMYB10-F | ACGCCACCACAAACGTCGTCG | qRT-PCR |
|  | MdMYB10-R | GGCGCATGATCTTGGCGACAGT |  |
| DQ341382 | 18S RNA-F | GTCACTACCTCCCCGTGTCA | qRT-PCR |
|  | 18S RNA-R | GAGCCTGAGAAACGGCTACC |  |
| CN944824 | MdCHS-F | GTGACTGTCCAGGAAGTTCGC | qRT-PCR |
|  | MdCHS-R | GCACACACTTGGATTCTCCTTTAG |  |
| CN946541 | MdCHI-F | GAAGGGTAAGACCGCCGAG | qRT-PCR |
|  | MdCHI-R | CACAATTCTCCGAAACTTTCTCAG |  |
| CN491664 | MdF3H-F | CGGGATGATGGGAAAACG | qRT-PCR |
|  | MdF3H-R | CGCTGGGTTCTGGAATGTG |  |
| CN491664 | MdF3’H-F | ACGATGGCGGATGTTACGG | qRT-PCR |
|  | MdF3’H-R | GCTTTGACCCTGCACTTGCT |  |
| AF117268 | MdDFR-F | GGACCCCGAGAATGAAGTG | qRT-PCR |
|  | MdDFR-R | CTCCACATTCACGGTTCCTG |  |
| AF117269 | MdANS-F | GAGAAGTATGCCAATGACCAGG | qRT-PCR |
|  | MdANS-R | GGCGGTTGCCTCAATGTAAT |  |
| AF119095 | MdFLS-F | ACGAGCAACCGGGAATCACAACTG | qRT-PCR |
|  | MdFLS-R | CCCAGTTGGAGCTGGCCTCAGTA |  |
| AF117267 | MdUFGT-F | GCTGACGAGTTGGGAGTGC | qRT-PCR |
|  | MdUFGT-R | CCTTCCGCTAAGTCTTTGATTC |  |
| DQ099803 | MdANR-F | GTTGCAACCCCTGTCAACTT | qRT-PCR |
|  | MdANR-R | CACGACCAAACCTGTTCCTT |  |
| DQ139836 | MdLAR-F | ACAACACCCACCCTTCTGAG | qRT-PCR |
|  | MdLAR-R | TGCAGCAAGGGCTAGTAGGT |  |
|  | MYB10-F | ATGGCCATGGAGGCCAGGAATTCATGGAGGGATATAACGAAAACC | MYB10 prey vector cloning |
|  | MYB10-F | GCAGCTCGAGCTCGATGGATCCCTATTCTTCTTTTGAATGATTCC |  |
|  | BiFCP1-F | GCGTCGACATGCCTGCAGGTCGACGA | BiFC vector cloning |
|  | BiFCP1-R | GCCCCGGGTGCCGCAAGAACCAACAC |  |
|  | BiFCP2-F | GCGTCGACATGGAGGAGTGCTCGACC |  |
|  | BiFCP1-R | GCCCCGGGTGCCGCAAGAACCAACAC |  |
|  | BiFCMYB-F | ATGGATCCATGGAGGGATATAACGAA |  |
|  | BiFCMYB-R | TTGTCGACTTCTTCTTTTGAATGATTC |  |
|  | YTHP1-F | GGGAATTCATGCCTGCAGGTCGAC | Y2H vector cloning |
|  | YTHP1-R | TACCCGGGGATCCTCTAGAGA |  |
|  | YTHP2-F | GGGGATCCTGGAGGAGTGCTCGAC |  |
|  | YTHP2-R | TCTATGCCGCAAGAACCAAC |  |
