# Supplementary material for: McMYB10 Modulates the Expression of a Ubiquitin Ligase, McCOP1 During Leaf Coloration in Crabapple
Source: Front Plant Sci. 2018 Jun 4;9:704. doi: 10.3389/fpls.2018.00704 (PMC5994411; doi:10.3389/fpls.2018.00704)
Supplement: Supplementary file 2 [file Table_2.DOCX]

**Supplementary Table S2.** Bait sequences used in this study.

| Bait sequences |  |
| --- | --- |
| MBS-box & Sp1-box | AACAAAAAATAATTACCCGCCTAACGTAGTAACG |
|  | TGGGCAGTTATAACAAAAAATAATTACCCGCCTA |
|  | ACGTAGTAACGTGGGCAGTTATAACAAAAAATAA |
|  | TTACCCGCCTAACGTAGTAACGTGGGCAGTTAT |
| Mutant | AACAAAAAATAATTAACCGATTAACGTAGTAACG |
|  | TGGGCTCATATAACAAAAAATAATTAACCGATTA |
|  | ACGTAGTAACGTGGGCTCATATAACAAAAAATAA |
|  | TTAACCGATTAACGTAGTAACGTGGGCTCATAT |
