# Supplementary material for: McMYB10 Modulates the Expression of a Ubiquitin Ligase, McCOP1 During Leaf Coloration in Crabapple
Source: Front Plant Sci. 2018 Jun 4;9:704. doi: 10.3389/fpls.2018.00704 (PMC5994411; doi:10.3389/fpls.2018.00704)
Supplement: Supplementary file 3 [file Table_3.DOCX]

**Supplementary Table S3.** Probe sequences used in this study.

| **MYB10 Binding Site** |  |
| --- | --- |
| Probe | GCTCCTCCCAAATTTT TGGGCAGTTATCCATGGGTGGTGCGCTGG |
| mProbe | GCTCATCTCAAATTTT TGGGTGCCAATCCATGATTGGTTAGCTGG |
