# Supplementary material for: McMYB10 Modulates the Expression of a Ubiquitin Ligase, McCOP1 During Leaf Coloration in Crabapple
Source: Front Plant Sci. 2018 Jun 4;9:704. doi: 10.3389/fpls.2018.00704 (PMC5994411; doi:10.3389/fpls.2018.00704)
Supplement: Supplementary file 4 [file Table_4.DOC]

**Supplementary Table S4.** Correlation analysis between the transcription of the *McCOP1* and *McMYB10* in crabapple leaves during different development stages.

|  | **‘Royalty’** | | **‘Flame’** | |
| --- | --- | --- | --- | --- |
|  | ***McCOP1-1*** | ***McCOP1-2*** | ***McCOP1-1*** | ***McCOP1-2*** |
| ***McMYB10*** | **0.784** | **0.799** | **0.906*** | **0.835** |
