# Supplementary material for: McMYB10 Modulates the Expression of a Ubiquitin Ligase, McCOP1 During Leaf Coloration in Crabapple
Source: Front Plant Sci. 2018 Jun 4;9:704. doi: 10.3389/fpls.2018.00704 (PMC5994411; doi:10.3389/fpls.2018.00704)
Supplement: Supplementary file 5 [file Table_5.DOCX]

**Supplementary Table S5.** Correlation analysis between the transcription of the *McCOP1* and *McMYB10* in crabapple leaves during a day.

|  |  |  | ***McCOP1-1*** | ***McCOP1-2*** |
| --- | --- | --- | --- | --- |
| **Young** | **Light** | ***McMYB10*** | **0.86** | **0.883*** |
|  | **Dark** | ***McMYB10*** | **0.901*** | **0.205** |
|  |  |  | ***McCOP1-1*** | ***McCOP1-2*** |
| **Mature** | **Light** | ***McMYB10*** | **0.635** | **0.737** |
|  | **Dark** | ***McMYB10*** | **0.578** | **0.256** |
