# Supplementary material for: McMYB10 Modulates the Expression of a Ubiquitin Ligase, McCOP1 During Leaf Coloration in Crabapple
Source: Front Plant Sci. 2018 Jun 4;9:704. doi: 10.3389/fpls.2018.00704 (PMC5994411; doi:10.3389/fpls.2018.00704)

**Supplementary Figure S1.** Protein interaction between McCOP1s and McMYB10. **(A)** Yeast two-hybrid assays (Y2H) were conducted with selective medium and blue color development. **(B)** BiFC assays in tobacco leaves.

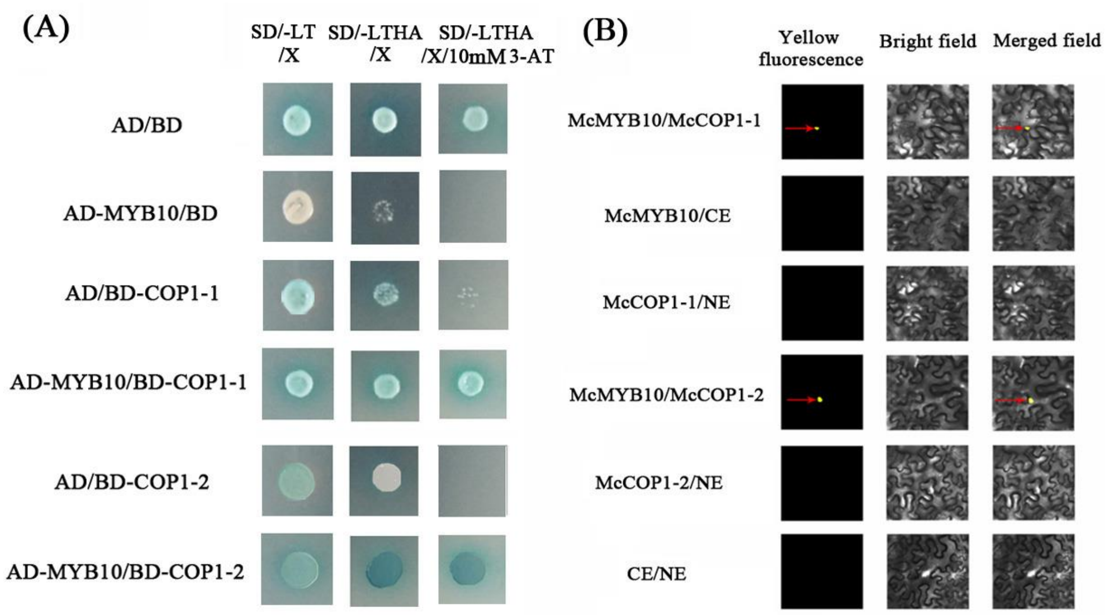

Supplement: Supplementary file 6 [file Image_1.PDF]
