# Supplementary figures and images for: McMYB10 Modulates the Expression of a Ubiquitin Ligase, McCOP1 During Leaf Coloration in Crabapple
Source: Front Plant Sci. 2018 Jun 4;9:704. doi: 10.3389/fpls.2018.00704 (PMC5994411; doi:10.3389/fpls.2018.00704)

**Supplementary Figure S2.** Sequence alignment of the *McCOP1-1* and *McCOP1-2* promoters

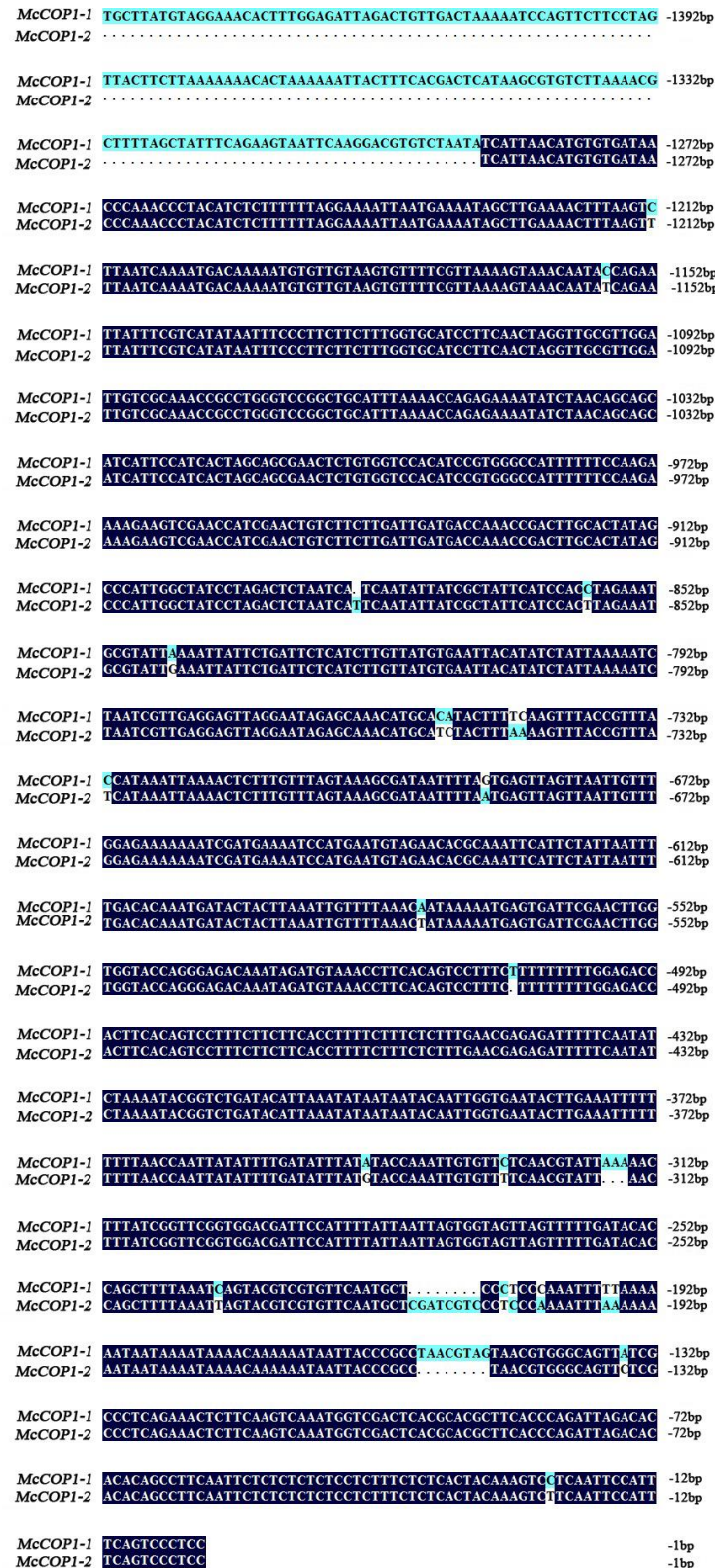

Supplement: Supplementary file 7 [file Image_2.PDF]
